# New Cognitive Deep-Learning CAPTCHA

**DOI:** 10.3390/s23042338

**Published:** 2023-02-20

**Authors:** Nghia Dinh Trong, Thien Ho Huong, Vinh Truong Hoang

**Affiliations:** 1 Department of Computer Science, Faculty of Electrical Engineering and Computer Science, VSB-Technical University of Ostrava, 17. Listopadu 15/2172, 708 33 Ostrava, Czech Republic; 2 Faculty of Information Technology, Ho Chi Minh City Open University, 97 Vo Van Tan Street, Ho Chi Minh City 722000, Vietnam

**Keywords:** security, cognitive, CAPTCHA, deep learning

## Abstract

CAPTCHA (Completely Automated Public Turing test to tell Computers and Humans Apart), or HIP (Human Interactive Proof), has long been utilized to avoid bots manipulating web services. Over the years, various CAPTCHAs have been presented, primarily to enhance security and usability against new bots and cybercriminals carrying out destructive actions. Nevertheless, automated attacks supported by ML (Machine Learning), CNN (Convolutional Neural Network), and DNN (Deep Neural Network) have successfully broken all common conventional schemes, including text- and image-based CAPTCHAs. CNN/DNN have recently been shown to be extremely vulnerable to adversarial examples, which can consistently deceive neural networks by introducing noise that humans are incapable of detecting. In this study, the authors improve the security for CAPTCHA design by combining text-based, image-based, and cognitive CAPTCHA characteristics and applying adversarial examples and neural style transfer. Comprehend usability and security assessments are performed to evaluate the efficacy of the improvement in CAPTCHA. The results show that the proposed CAPTCHA outperforms standard CAPTCHAs in terms of security while remaining usable. Our work makes two major contributions: first, we show that the combination of deep learning and cognition can significantly improve the security of image-based and text-based CAPTCHAs; and second, we suggest a promising direction for designing CAPTCHAs with the concept of the proposed CAPTCHA.

## 1. Introduction

CAPTCHA (Completely Automated Public Turing test to tell Computers and Humans Apart), or HIP (Human Interactive Proof), is a standard security feature that determines whether a user is a computer program or a human. It has a wide variety of applications such as: free email services, online polls, worms, and spam, preventing dictionary attacks and cyber-attacks [1], etc. Generally, CAPTCHA is a cryptographic procedure [2] with an underlying difficulty assumption with an AI problem behind it. CAPTCHA indicates a win–win strategy: either the CAPTCHA is not defeated, and a method for distinguishing people from machines exists; or the CAPTCHA is broken, and a difficult AI issue has been resolved.

Text-based CAPTCHAs were the original type and continue to be the most-used CAPTCHA technique. To enhance the security of text-based CAPTCHAs, developers used various techniques such as distortion, noise, rotation, etc. However, as deep-learning approaches, enhanced algorithms, and advanced hardware devices have been developed, text-based CAPTCHAs have been demonstrated to be less secure. The recent failure of most text-based CAPTCHAs raises security and accessibility concerns. As a result, rather than character recognition, more designs are focusing on image-based recognition. However, proposed image-based CAPTCHAs can be bypassed by deep-learning techniques, such as image classification and object detection. Lately, several researchers have discovered that some state-of-the-art deep neural networks, known as adversarial examples, can be deceived by introducing some modest alterations that humans do not notice in the original image [3]. Furthermore, CAPTCHA techniques based on cognitive ability have largely replaced traditional CAPTCHA approaches, providing improved security. Cognition refers to brain-based abilities that are the result of a unique combination of neurobiological and psychological techniques. These CAPTCHA approaches distinguish humans from automated bots by using physical (something you have), biometric (something you are), and knowledge-based (something you know) factors. However, they are vulnerable to human-assisted relay attacks because they have limited challenges and can only be completed through secure devices. Throughout the fight against security assaults, cognitive-based CAPTCHA methods clearly have a competitive advantage over other systems.

In our research, we introduce a CAPTCHA called zxCAPTCHA, mixing image-based, text-based, and cognitive-based CAPTCHA schemes’ characteristics and applying adversarial examples, neural style transfer, and some defense deep-learning techniques, to improve the security of the CATCHA. Unlike previous research into CAPTCHA in which designers only offered changes by adding distortion, artificial noise, or a complex background, deep-learning techniques and cognitive characteristics are used in the proposed CAPTCHA. By performing pixel updates on images fused with another style image, we use neural style transfer [4] to increase the variety of the image database and the difficulty of machine classification. Furthermore, applied adversarial examples can deceive neural networks by introducing small perturbations in original images that are imperceptible to humans. In zxCAPTCHA, a user is required to select all corresponding images in the correct order based on the content of a text-based CAPTCHA and their selected stylized topic. Our work makes two major contributions:

- First, we show that the combination between deep learning and cognition can significantly improve the security of image-based and text-based CAPTCHAs.
- Second, we suggest a promising direction for designing CAPTCHAs. The proposed CAPTCHA can be varied for different cognitive CAPTCHA schemes by changing their attributes. Specifically, the attribute of text group order can be natural order, inverse order, or special order, such that any text group with special characteristics can be requested to be picked up with higher priority. Furthermore, the background images and text can be localized to make them more familiar to users in their local surroundings. As a result, we can see that the use of this CAPTCHA is widespread and simple to adapt to any system that requires CAPTCHA protection against automated bots.

The remainder of the paper is organized as follows. Section 2 contains a summary of related work. Section 3 introduces our proposed CAPTCHA system. Section 4 describes the techniques used in the CAPTCHA. Section 5 goes over the implementation. Section 6 presents the experiment results and evaluates the usability and security of this proposal. Section 7 concludes with closing remarks and research directions for the future.

## 2. Related Works

The most extensively used CAPTCHA system has been text-based CAPTCHA. CAPTCHA creators attempted to improve security by adding different resistance methods to current text CAPTCHAs, such as noise, backdrops, crowding characters together (CCT), etc. However, as studies [5,6,7,8] have shown, all these resistance mechanisms appear to be ineffective. Many research communities are concentrated on building attack methods for existing text-based CAPTCHAs. Early attempts to attack numerous CAPTCHAs used shape characteristics, distortion estimation, and machine-learning techniques. Recent research papers [9] offered generic approaches to attack text-based CAPTCHAs. Deep-learning techniques have lately been used as a new approach to overcome a CAPTCHA. Goodfellow et al. [10] used CNNs to solve reCAPTCHA with a 99.8% success rate. These successful attacks show that text-based CAPTCHAs are no longer considered extremely secure.

Chew and Tygar [11] were pioneers of applying image recognition to create CAPTCHA. Since then, many new image-based CAPTCHAs have been proposed. Image-based CAPTCHAs are classified into three types: image-selecting CAPTCHA, mouse-dragging CAPTCHA, and clicking CAPTCHA. Image-selection CAPTCHAs often require users to select numerous images from a set of photographs based on a certain description. Typically, image-selection CAPTCHA instances are ASIRRA CAPTCHA, Facebook CAPTCHA and Google CAPTCHA, IMAGINATION [12], FR-CAPTCHA [13], ARTiFACIAL CAPTCHA [14], etc. To classify these images, the most widely used and effective methods for cracking image-based CAPTCHAs have been to train neural networks, with outstanding research by Cheung [15], Sivakorn et al. [16], Zhu et al. [17], Gao et al. [18], Li [19], Srivastava et al. [20], Fatmah et al. [21], etc. Mouse-based CAPTCHAs require users to drag an image fragment back to its original location or adjust an image’s orientation, such as: What’s up CAPTCHA [22], Capy CAPTCHA [23], and Geetest CAPTCHA [24]. By employing a low-cost side-channel approach, Hernández-Castro et al. [25] successfully overcame Capy CAPTCHA. In general, there still exist security issues making image-based CAPTCHAs vulnerable to automated attacks. Zhang et al. [26] investigated the impact of adversarial examples on CAPTCHA security (including image-based and text-based CAPTCHAs). They generated adversarial CAPTCHAs directly using current generation algorithms of adversarial examples. Their experimental findings show that adversarial examples improve the robustness of CAPTCHAs. DeepCAPTCHA, a novel image-based CAPTCHA technique developed by Osadchy et al. [27], introduces immutable adversarial noise (IAN) deceiving deep-learning techniques. The authors [28] developed a GAN-based technique to break text-based CAPTCHAs by applying transfer learning to fine-tune the base solution on synthetic CAPTCHAs. Although this strategy can lower the cost of an attack by classifying data, it is still sensitive to adversarial CAPTCHAs. In conclusion, while text-based and image-based CAPTCHA schemes remain vulnerable to automated attacks, adversarial examples and other deep-learning techniques demonstrate the effectiveness and robustness of these CAPTCHA schemes when applied.

Traditional CAPTCHA schemes have mainly been superseded by cognitive CAPTCHA approaches based on cognitive abilities that give increased security. Cognitive abilities are brain-based capabilities resulting from a unique blend of neurobiological and psychological strategies. Typically, cognitive-based CAPTCHA instances are BeCAPTCHA-Mouse [29], Gametics [30], reCAPTCHA V2, Invisible reCAPTCHA, CAPPCHA [31], Sensor CAPTCHA [32], Pedometric CAPTCHA [33], CAPTCHAs by Mantri et al. [34] and Frank et al. [35], Unseen CAPPCHA [36], AccCAPTCHA [37], GISCHA [38], Ababtain et al. [39], SenCAPTCHA [40], BrightPass [41], PIN-based authentication CAPTCHA [42,43], Match-the-Sound CAPTCHA [44], etc. In conclusion, practically all cognitive-based CAPTCHAs with sensor assistance have an advantage over competing strategies in that they have not yet been penetrated by automated attacks. This will lead to a very positive future for CAPTCHA design.

In this paper, we will discuss a new proposed CAPTCHA and its robustness that leverages the important characteristics of image-based, text-based, and cognitive-based CAPTCHA schemes along with deep-learning techniques such as neural style transfer and adversarial examples.

## 3. Proposed CAPTCHA System

### 3.1. Design Concept

**Figure 1:**
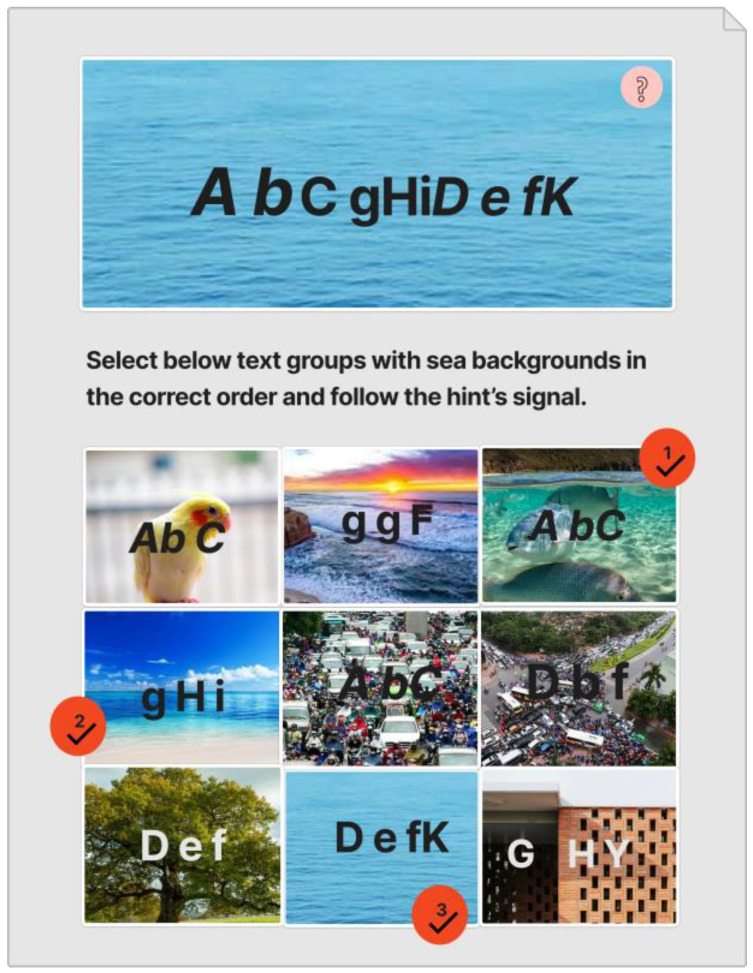
zxCAPTCHA design.

Inspired by [45,46,47], this CAPTCHA has two layers of protection, presented in Figure 1. The first layer of protection is a text-based CAPTCHA of three groups of text with each group ranging from three to five characters in length. The second layer is a CAPTCHA based on selection, in which users must select corresponding images, containing relevant text groups, with the mentioned topic background in the correct order and follow the hints. The hint symbol, a pink circle with a question mark inside, located in the top-right corner of the text-based CAPTCHA, guides the user when it is necessary to select a correct image or an incorrect image by rendering random colors, such as red for the incorrect image and blue for the correct image. Users can obtain status information by clicking on the hint symbol.

In text-based CAPTCHA, text groups have been used in a variety of techniques to enhance the security of the CAPTCHA, for example: distortion, rotation, occluding lines, adding artificial noise, or complex background, etc. The distorted text can be smoothly merged to the topic background using seamless cloning [48]. Then, the merged image is mixed with a random style image by neural style transfer [4,49] to make it difficult to be recognized by automated bots. Furthermore, the resultant image applies adversarial examples [50] and some defense methods to protect the security against CNN/DNN attacks.

There are nine images with varying content in selection-based CAPTCHA, some with correct text but incorrect backgrounds or vice versa. It generates many deliberate misleads that can fool automated bots. Again, each image in selection-based CAPTCHA employs the same techniques as in text-based CAPTCHA to make recognition and classification more difficult for automated bots. Furthermore, by requiring users to select correct or incorrect images based on hints, the CAPTCHA can test users’ cognitive ability, which can be extremely difficult for bots, and can avoid relay attacks.

### 3.2. CAPTCHA Architecture

**Figure 2:**
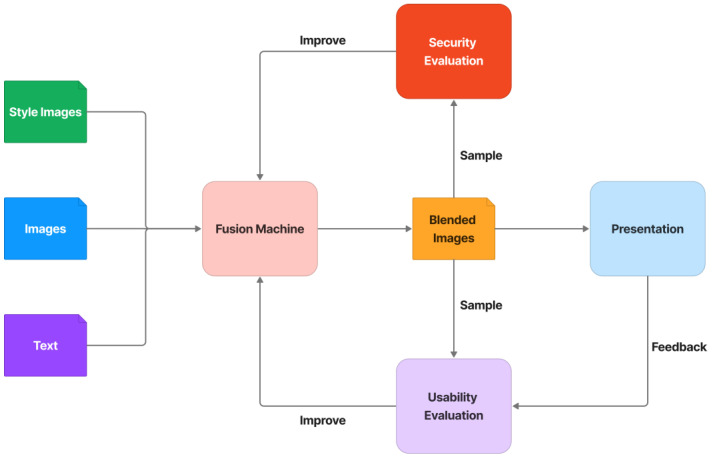
Logic CAPTCHA architecture.

The CAPTCHA architecture is made up of several major components, including a fusion machine, a security evaluation, and a usability evaluation, shown in Figure 2. Through seamless cloning, neural style transfer, adversarial examples, and defense methods, the fusion machine is in charge of blending text, images, and random style images. Some of the blended images are subjected to a security evaluation process to assess the CAPTCHA’s resistance to automated attacks. The security assessment concludes with some state-of-the-art DNN attacks that attempt to analyze the CAPTCHA for object classification. Useful information extracted from the security evaluation process is transferred to the fusion machine in order to improve the fusion process and make it more resistant to attacks. Furthermore, some of the blended samples or feedback challenges are transferred to the usability evaluation process to assess how easy it is to use this CAPTCHA, such as whether users can easily recognize and resolve this CAPTCHA or why users meet many difficulties in resolving it. Many testers participate in the usability evaluation process. They are tasked with resolving the challenge samples and providing recommendations to improve the usability of the CAPTCHA. These helpful suggestions are converted into relevant designs in the fusion machine, thereby increasing the usability of the CAPTCHA.

In general, this architecture provides CI/CD (Continuous Improvement and Continuous Delivery) of security and usability for the generation of CAPTCHA challenges. It allows the CAPTCHA to continue improving its security against automated bots by rotating the most advanced attacking algorithms while maintaining stable usability.

## 4. Applied Techniques

### 4.1. Neural Style Transfer

Neural style transfer is a deep-learning technique for producing aesthetic pictures [4,49]. By using a deep CNN network that has been trained, this technique may take the aesthetic of creative photos and apply it to other images. Using a VGG-19 [51] network with 16 convolutional and 5 pooling layers and without fully connected layers, Gatys et al. [4] were the first researchers to investigate the technique, proposing the neural-style-transfer architecture. The cost of creating a stylized image, however, is too high because each step of the process involves forward and backward propagation. Johnson et al. [49] and Ulyanov et al. [52] combined the benefits of optimization-based algorithms with feed-forward image transformation to outperform Gatys et al. in the iterative optimization process. Even though these techniques increase computational speed, a great deal of flexibility is lost because each new painting style requires a new style-transfer network to be developed and trained. To enable real-time stylization utilizing any content–style image pair, in this work we implement research [53] using fast style-transfer networks that avoid border patterns, caused by zero padding, and checkerboard patterning, caused by transposed convolutions.

The goal of neural style transfer [53] is to generate a stylized image y with a style similar to a style image $y_{s}$ and content similar to a content image $y_{c}$. The reconstruction procedure is divided into two steps: content reconstruction and style reconstruction. The style is the correlation between different filters (Gram matrices) in each layer, while the content is the CNN-extracted feature map. The differences between stylized and original images are measured using content loss and style loss. The algorithm proposes two similarity definitions in style and content:

- If the difference between the features’ Gram matrices has a small Frobenius norm, then the two images are stylistically similar.
- If the high-level features of two images, as determined by a trained classifier, are near in Euclidean distance, then the two images have similar content.

In the networks, the feature maps of y, $y_{c}$ and $y_{s}$ are denoted by $F^{l}$, $P^{l}$ and $S^{l}$ in layer l, respectively. $N_{l}$ is the layer’s filter number, and $M_{l}$ is the feature map’s spatial dimension. $F^{l}$, $P^{l}$ and $S^{l}$ are all kept in the same size matrix ${\mathbb{R}}^{\left\{N_{l}\times M_{l}\right\}}$. ***L*** and ***C*** are the number of style layers and content layers, respectively. The difference between target and output images in terms of content and style is measured using global loss $L_{total}$. In neural style transfer, the main goal is to reduce total loss $L_{total}$ to improve the output image’s texture and details. It is computed by:

(1) $$L_{total}=\alpha L_{content}+\beta L_{style}\;$$

where $L_{style}$ is the style loss calculated in Equation (3), $L_{content}$ is the content loss calculated in Equation (2), and **α** and ***β*** are weighting factors that balance the importance of content and style. $L_{content}$ is the Euclidean distance between the generated and content images:

(2) $$L_{content}\left(y,\;y_{c}\right)=\overset{C}{\underset{l=1}{\sum }}\frac{1}{N_{l}}\|\left(F^{l}-P^{l}\right){\|}_{2}^{2}\;$$

$L_{style}$ reflects the Gram matrices difference between the style image and generated image, and can be calculated as:

(3) $$L_{style}\left(y,y_{s}\right)=\overset{L}{\underset{l=1}{\sum }}\frac{1}{N_{l}}\|G^{l}-A^{l}{\|}_{F}^{2}$$

with $G^{l}$ and $A^{l}$ representing Gram matrices of style image and generated image associated with the layer-l activations.

**Figure 3:**
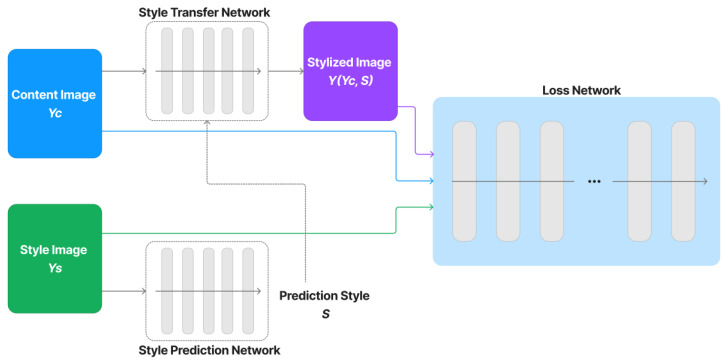
Overall network architecture.

Our network model, which is depicted in Figure 3, is primarily built for neural style transfer based on the study [53]. To transform photos into stylized images, a style-transfer network is trained with the support of vector ***S***, a collection of normalization constants, which is extracted from a style-prediction network. Using a loss network that has been trained particularly for image classification, we construct perceptual loss functions that measure perceptual differences in the style and content of images.

**Figure 4:**
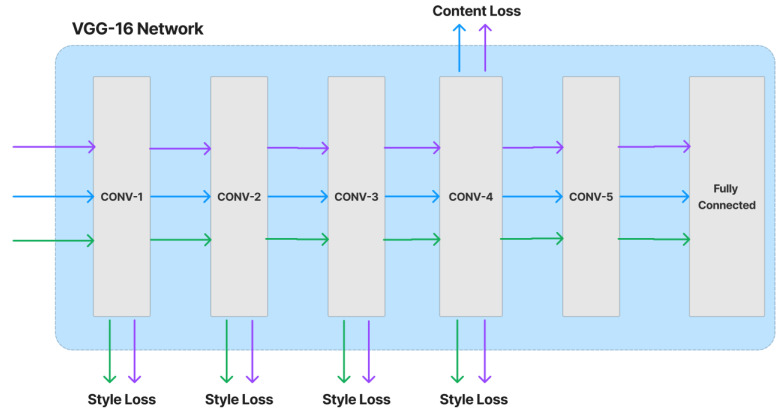
Loss network VGG-16.

**Figure 5:**
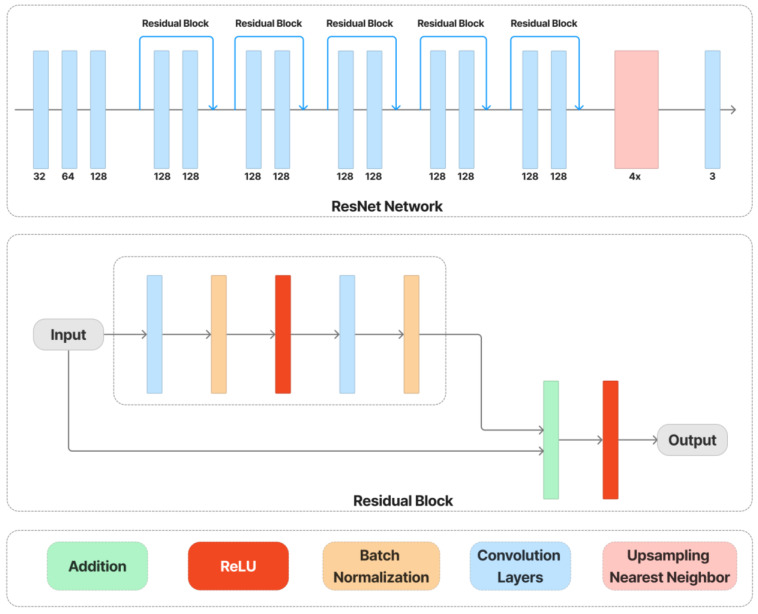
Style-transfer network.

**Figure 6:**
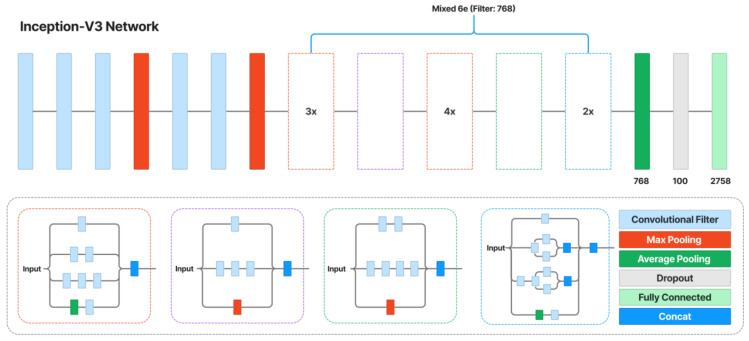
Style-prediction network.

The content and style loss network are obtained from a VGG image classification network [51], typically VGG-16, shown in Figure 4. The network of style transfer, shown in Figure 5, is mainly as follows [54]. Strided and fractionally strided convolutions are used for in-network downsampling and upsampling instead of pooling layers. Using the architecture of [55], our network body is made up of five residual blocks [56]. Except for the output layer, all non-residual convolutional layers are followed by spatial batch normalization [57] and ReLU nonlinearities to make sure that the output image has pixels in the [0,255] range. Other than the first and last layers, which use 9 × 9 kernels, all convolutional layers use 3 × 3 kernels. Additionally, mirror padding is used in place of zero padding, and transposed convolutions (also known as deconvolutions) are substituted by nearest-neighbor upsampling before a convolution. By using mirror padding, border patterns that might occasionally result from zero padding in SAME-padded convolutions are avoided, while the substitution for transposed convolutions eliminates checkerboard patterning. In Figure 6, the applied style-prediction network, which mostly follows the Inception-v3 design [58], predicts an embedding vector S from an input style image and gives the style-transfer network a set of normalization constants. We calculate the mean of all activation channels in the Mixed-6e layer, and this produces a feature vector with a 768-dimensional output. To predict the final embedding S, we then apply two fully connected layers on top of it with filters 100 and 2758 (the last Softmax layer is removed). The key benefit of this strategy is that the model may generalize to an image with an unknown style by predicting its appropriate style embedding at runtime.

Our network model consists of three core networks: a loss network, a style-prediction network, and a style-transfer network. By replacing the internal network models with modern models or adjusting their parameters, there are still plenty of opportunities to improve the model performance and efficacy. These activities fall outside the purview of this study. Here, we concentrate more on how to efficiently develop zxCAPTCHA to guard against DNN/CNN automated bots.

### 4.2. Adversarial Examples

The authors in [3] first introduced the idea of adversarial examples in 2014. Modern neural networks and other machine-learning models were discovered to be susceptible to small, almost imperceptible changes in the original images. In other words, these machine-learning algorithms incorrectly identify cases that are little different from those that are correctly classified. In addition, adversarial examples that have an impact on one model frequently also have an impact on another model, even if the two models have different architectures or were trained using various training sets, if both models were trained to complete the identical job. They possess both fooling and transferability properties.

Numerous new strategies for generating adversarial examples have also been proposed because of extensive study of adversarial examples. The Fast Gradient Sign Method (FGSM), which Goodfellow et al. [50] proposed in 2014, can be used to construct adversarial examples using the gradient descent process. Seyed–Mohsen Moosavi-Dezfooli proposed DeepFool [59] in 2016, which computes a minimal norm of adversarial perturbation for a given image in an iterative manner. Even in the real world, adversarial examples can pose a security risk to neural networks, as Alexey Kurakin et al. [60] showed. To calculate adversarial examples, they also suggested the Basic Iterative Method (BIM). The technique iteratively applied the quick gradient sign approach, but it produced a larger fooling ratio. Additionally, they improved this method and proposed the Target Class Gradient Sign Method (TGSM) and Iterative Least Likely Class Method (ILLCM). To find a single perturbation vector that can trick networks with many natural images, Moosavi-Dezfooli et al. introduced the Universal Adversarial Perturbations Method (UAPM) [61] in 2017. This method computes independent quasi-imperceptible universal perturbation vectors. Sarkar et al. [62] proposed two black-box attack algorithms, UPSET and ANGRI. Targeted fooling is accomplished using the perturbations brought on by both ANGRI and UPSET. To create robust adversarial perturbations, Ivan Evtimov et al. [63] presented the Robust Physical Perturbations (RP2) technique. The approach obtained high attack success rates in the real world while considering a variety of physical circumstances. A Jacobian-based Saliency Map Algorithm (JSMA) was suggested in [64] for iteratively creating an adversarial example. Adversarial Transformation Networks (ATNs) were trained by Baluja and Fischer [65] to provide adversarial examples against other targeted networks or groups of networks. An adversarial patch was suggested by Google researchers [66]. For some real-world intelligent devices, adversarial examples pose possible security risks. The remedies were sought by certain researchers. Gu and Rigazio [67] developed a model in 2015 that can withstand adversarial examples, but they were still unable to eliminate their impact. Warren He [68] made an attempt with five defenses for adversarial examples, but even ensembles of weak defenses were shown to be ineffective. In 2017, Xie [69] demonstrated that the impact of adversarial examples is diminished by random scaling. Additionally, adversarial examples with random padding will lower the network’s fooling rate. Jiajun Lu [70] recently asserted that adversarial examples cannot always ensure that they will trick the neural network for large photographs shot from various distances and angles. OpenAI [71], however, instantly said on their official blog that they created images that, when viewed from various perspectives and distances, can reliably trick automated bots’ classification. In the foreseeable future, research into adversarial examples in neural networks will continue to be an exciting topic.

In this study, we use the Fast Gradient Sign Method (FGSM) and Backward Pass Differentiable Approximation (BPDA) adversarial generation techniques for non-targeted attacks that result in misclassification to a non-specific class. If FGSM is used in black-box settings, where hackers cannot connect the target networks’ gradients, BPDA is used in white-box settings, where attackers can access the target networks’ gradients and fully understand the defense that is being utilized. We are able to assess the CAPTCHA’s robustness and resilience against automated bots due to research into two different setting methods.

#### 4.2.1. FGSM Generation Method

With the greatest benefits of speed and ease, FGSM generates an adversarial example using the gradients of the neural network. The method produces a new image, called the adversarial image, which maximizes the loss for an input image by using the gradients of the loss with respect to the input image. This can be summarized using the following expression:

(4) $$x^{\prime }=x+\epsilon \times sign\left(\delta_{x}J\left(\theta ,\;x,\;y\right)\right)\;$$

where ***x′*** is an adversarial image, ***x*** is original input image, ***y*** is original input label, sign()  is a function to obtain the sign of the gradient direction, ϵ guarantees small perturbations, θ is a training model’s set parameters, and ***J*** is the loss function. The goal of maximizing the loss function ***J*** is to ensure that the difference between the output picture and the original input image is as small as possible while still increasing the likelihood that an output image from an input image would be classified incorrectly. The FGSM process is as follows:

- Forward propagation is used to obtain the expected labels.
- The gradient direction is set to reduce the likelihood that the label is true.
- The backward propagation modifies the weight parameters.
- The adversarial examples are recomputed using forward propagation with the modified weights.

#### 4.2.2. BPDA Generation Method

In the proposal [72], BPDA is suggested for situations in which it is difficult or impossible to compute the gradient of a preprocessor that is used as defense. Utilizing this technique, we can approximate the gradient of non-differentiable layers to attack non-differentiable networks. The gradient is estimated by computing the forward pass normally but replacing a non-differentiable layer f(.) with a differentiable approximation g(.)=f(.)  on the backward pass. In particular, the denoising defense can be seen as the neural network’s initial layer, which handles input pre-processing. When this preprocessing is differentiable, conventional attacks can be applied. When it is impossible to compute the gradient of the pre-processing, Athalye et al. [72] proposed to approximate it using the identity function in which the pre-processing step computes a function g(x)≈x . This can be summarized using the following expression:

(5) $$x^{\prime }=clip\left(x+\epsilon \times sign\left(\delta_{x}J\left(\theta ,\;g\left(x\right),\;y\right)\right)\right)$$

where ***x’*** is an adversarial image, ***x*** is an original input image, ***y*** is an original input label, sign()  is a function to obtain the sign of the gradient direction, ϵ guarantees small perturbations, θ is a training model’s set parameters, and ***J*** is the loss function. We use a FGSM framework to implement this logic, which leads to the following process when applied to a raw image x:

- Use any of the defense techniques to denoise x and obtain denoise(x).
- Forward propagate denoise(x) through the net and compute its loss.
- Calculate the gradient $\delta_{x}J\left(\theta ,\;denoise\left(x\right),\;y\right)\;$ of the loss from the denoised image.

#### 4.2.3. Generation Networks

Two networks are employed in our study to generate adversarial examples: ResNet-101 [56] and VGG-16 [51]. VGG-16 is a CNN (Convolutional Neural Network) with 16 layers that is widely regarded as one of the best computer-vision models available to date. This model took first and second places in the 2014 ILSVRC challenge. ResNet, which stands for Residual Network, is a type of CNN introduced in 2015 by He Kaiming et al. [20]. ResNet-101 has 101 layers and was created using convolution neural networks and residual blocks. This network took first place in the ILSVRC 2015 classification task.

### 4.3. Text Distortion Optimization

**Figure 7:**
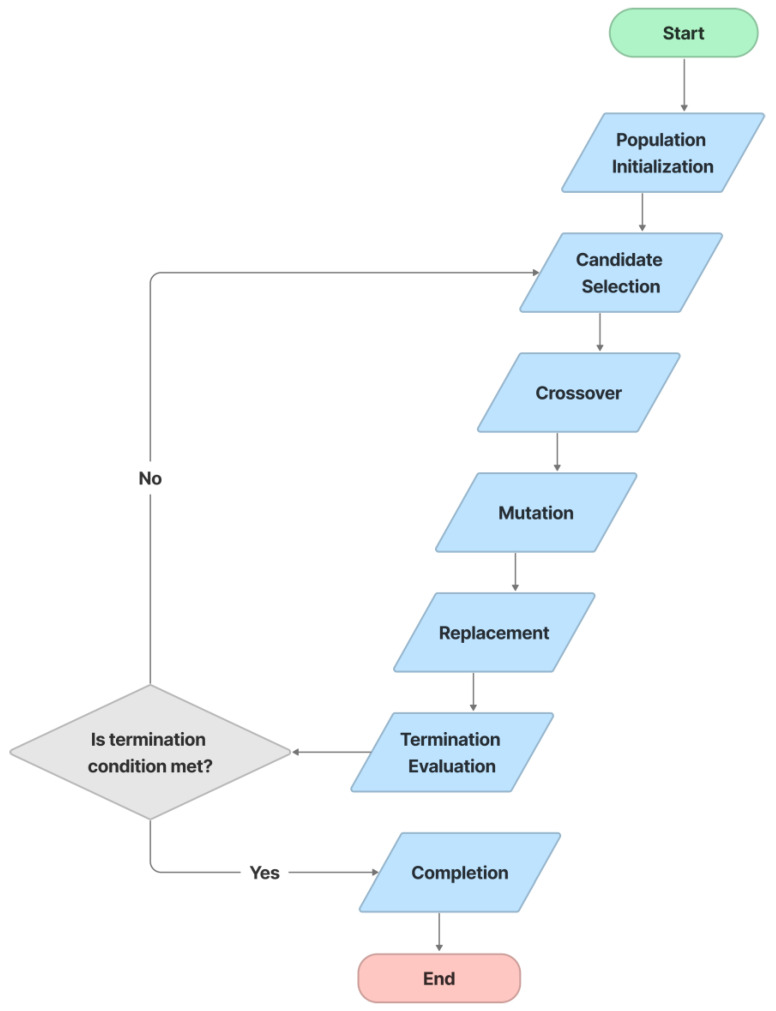
GA optimization steps.

In this study, text distortion is improved using a Genetic Algorithm (GA) [73]. GAs are search-based algorithms based on natural selection and genetics, and they are a subset of the much larger branch of computation known as Evolutionary Computation. Typically, we have a population of possible text distortion types. These distortion types use recombination and mutation (as in natural genetics) to produce new children, and the process is repeated over multiple generations. Everyone (distortion type) is given a fitness value, and the fitter individuals have a better chance of mating and producing more fitter individuals. This is consistent with Darwin’s theory of “Survival of the Fittest”. In this strategy, we continue to evolve better individuals over generations until we reach a termination point. In Figure 7, the process of text distortion optimization includes the following steps: population initialization, candidate selection, crossover, mutation, replacement, termination evaluation, and completion.

#### 4.3.1. Chromosome Initialization

A chromosome contains many genes. Each gene is represented by a distortion type with an intensity value that falls within a certain range. A chromosome in this study contains two to three genes. Depending on the type of deformation and the intensity value, each gene has a fitness value:

(6) $$f^{i}=level^{i}+\frac{value^{i}-min^{i}}{max^{i}-min^{i}}$$

where $f^{i}$ is the fitness value of a gene ***i*th**; $level^{i}$ is the level score of the gene, reflecting how a distortion type with values ranging from one to three affects the CAPTCHA; $value^{i}$ is the intensity value of the gene; $max^{i}$ is the maximum intensity value, and  $min^{i}$ is the minimum intensity value. This algorithm randomly generates ten chromosomes. Each chromosome selects two to three distortion types from the extensible distortion list, Table 1, and assigns random extensity values within their ranges. The fitness value of each chromosome is the sum of the fitness values of their genes, as calculated by Equation (6).

#### 4.3.2. Candidate Selection

In this process, we used a roulette wheel to select parent candidates for the next generation. Chromosomes are selected at a proportional rate to their fitness:

(7) $$p_{i}=\frac{\alpha^{i}}{\sum_{i=0}^{n}\alpha^{i}}$$

where $p_{i}$ is the probability of selected chromosome ***i*th**, $\alpha^{i}$ is the fitness of chromosome ***i*th**, and n is the total number of chromosomes. The procedure is as follows:

- Determine ***S***, which is the sum of fitness.
- Generate a random number between ***0*** and ***S***.
- Sort the population in descending order of chromosome fitness.
- Starting at the top of the population, add the fitness to the partial sum ***P*** until ***P < S***.
- The chosen individual is the one for which ***P*** exceeds ***S***.

#### 4.3.3. Crossover

Approximately 80% of the parent chromosomes in the candidate-selection process are chosen at random. Two child chromosomes are created by combining values from two parent chromosomes. The Whole Arithmetic Recombination method is employed in this process by using the following formula to recombine two genes of the same type (same distortion type) in two parent chromosomes:

(8) $$c_{1}=\omega \times a_{1}+\left(1-\omega \right)\times a_{2}$$

(9) $$c_{2}=\omega \times a_{2}+\left(1-\omega \right)\times a_{1}$$

where ω is the coefficient with value 0.25, $c_{1}$ and $c_{2}$ are extensity values of child 1 and child 2, and $a_{1}$ and $a_{2}$ are extensity values of parent 1 and parent 2, respectively.

#### 4.3.4. Mutation

To avoid stagnation at local optima, approximately 5% of chromosomes are mutated. This ensures that the entire solution space, rather than only values close to those of the parent chromosomes, is searched. The following formula is used to evaluate the values of several genes on a mutation chromosome:

(10) $$m=\frac{c+g}{2}\;$$

where ***m*** is the mutation value of a gene, ***g*** is a random value within their extensity range, and ***c*** is the extensity value of the gene.

#### 4.3.5. Replacement

The ***λ + μ***-update replacement process is used to determine which chromosomes will be kept in a new generation of chromosomes. The chromosomes with the highest fitness values from both child generations and their parents are kept in this method to save good chromosomes.

#### 4.3.6. Termination Evaluation

Following replacement, all fitness values in chromosomes are compared, and the best fitness value for each generation is recorded. The genetic-learning algorithm can be terminated once enough generations, or the best fitness value, has been reached. Otherwise, the GA algorithm continues to run, and to generate new generations, the chromosomes obtained from the replacement process are fed into the selection step. The termination condition is depicted below. By retaining the chromosomes with the highest fitness values from both the parent and child generations, this strategy saves good chromosomes from the parent generation that would otherwise be lost due to standard generational replacement.

(11) $$condition=\{\begin{array}{c} done\;if\;g\geq 10\\ done\;if\;best_{g}<1.01\times best_{g-5}\\ continue\;otherwise \end{array}$$

where g is the number of generations, $best_{g}$ is the highest fitness in the generation ***g***, and $best_{g-5}$ is the highest fitness in the generation (***g*** − 5).

#### 4.3.7. Completion

When the termination criteria are met, the genetic-learning process is terminated. Chromosomes with best fitness will be used in the CAPTCHA generation.

## 5. Implementation

### 5.1. Datasets

The CAPTCHA uses the extended Modified National Institute of Standards and Technology database (EMNIST) [74] with the datasets provided below:

- ByClass
- ByMerge
- Balanced
- Letters
- Digits
- MNIST

For images, we employ ImageNet ILSVRC-2012 [75], which was used for the ImageNet large-scale visual recognition challenge in 2012.

### 5.2. Text-Based Adversarial CAPTCHA

**Figure 8:**
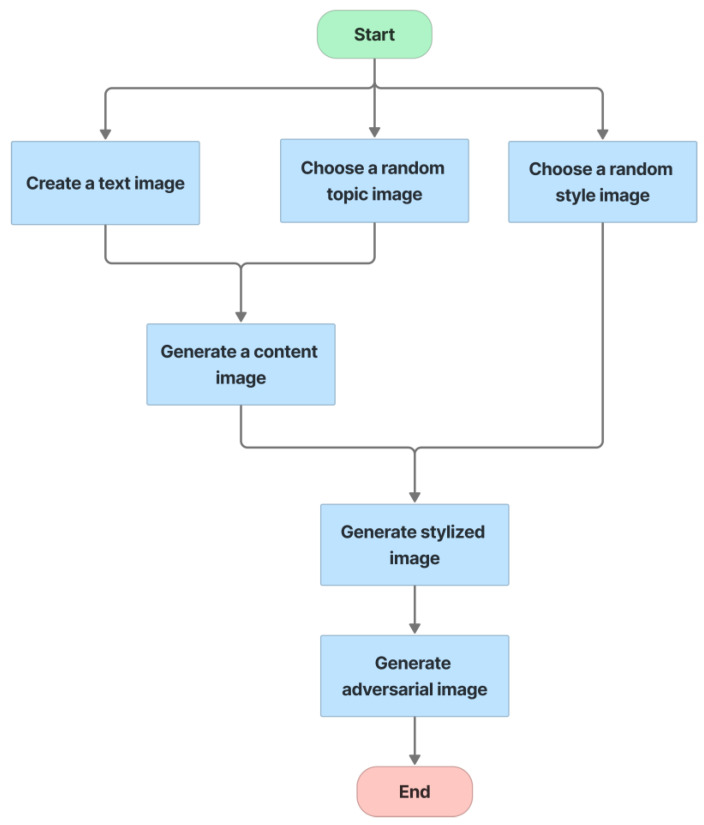
Text-based CAPTCHA generation.

The text-based CAPTCHA consists of three groups of text, each with three to five random characters drawn from the EMNIST dataset. The CAPTCHA-generation process is illustrated in Figure 8, as follows:

- Step 1, text image creation: for each group, select three to five letters at random from EMNIST. The characters of each group are distorted [4.3], and parameter ***O*** is used to control the overlap between two characters, with the default value for ***O*** being 0. The text from three groups are then concatenated to form a whole CAPTCHA with a white background. The size of a whole text-based CAPTCHA is 256 × 768.
- Step 2, topic image selection: from the ImageNet dataset, choose a random image of the global topic, called the topic image.
- Step 3, style image selection: from the ImageNet dataset, choose a random image for the style image.
- Step 4, fusion: by using seamless cloning [48], the fusion machine merges the text image with the topic image to create the content image. This content image is then style transferred from the style image by the neural-style-transfer method. Finally, the stylized image is transformed into an adversarial example to cause misclassification of the CNN/DNN networks.

### 5.3. Grid-Based Adversarial CAPTCHA

**Figure 9:**
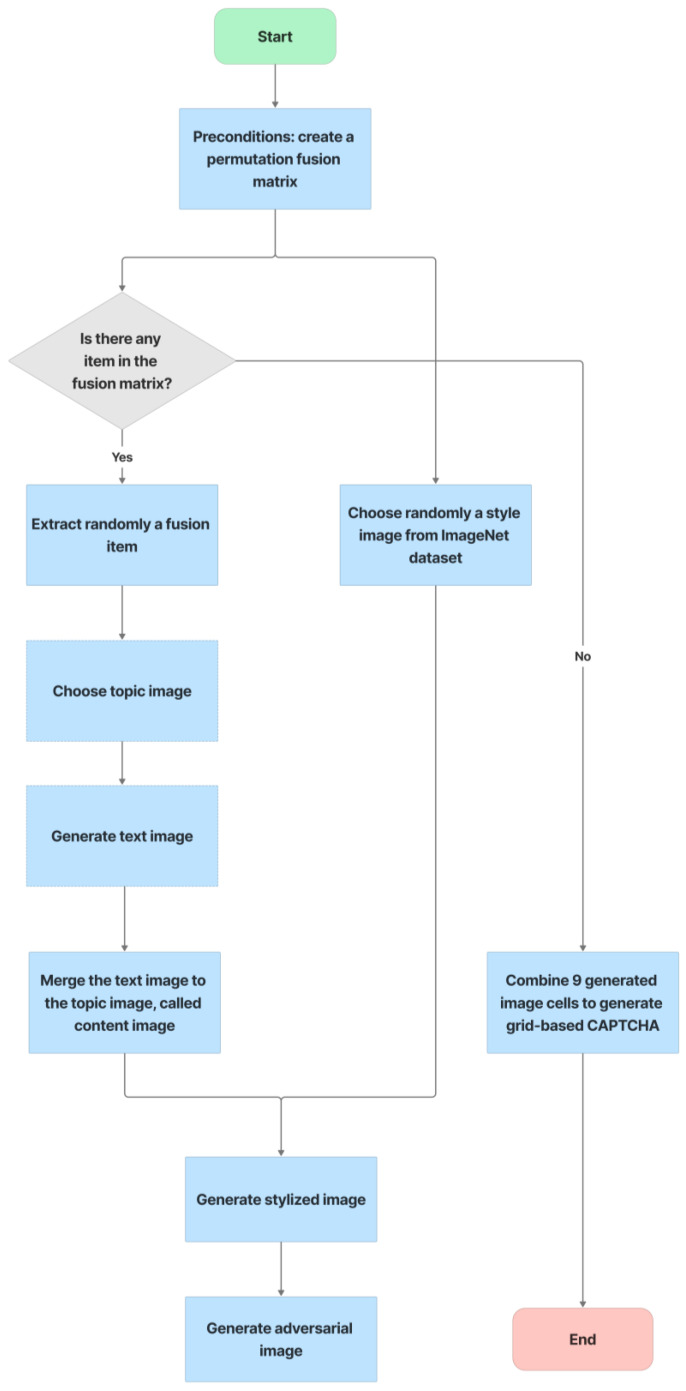
Grid-based CAPTCHA generation.

With six incorrect cells and three correct cells, the grid-based CAPTCHA is made up of nine image cells, each with correct or incorrect text in terms of the text groups of the text-based CAPTCHA and correct or incorrect background in terms of the global topic. Users must select the correct or incorrect image cells based on a correct order and the hint symbol’s signal. Figure 9 depicts the generation process as follows:

- Preconditions: set the grid-based CAPTCHA size to nine and create a Boolean permutation fusion matrix. The fusion matrix contains nine status pairs reflecting the correct or incorrect topic background and the correct or incorrect group text, with three correct fusion items (correct topic background and correct group text).
- Each fusion item is extracted from the matrix. To obtain the cell topic image, a random image of a correct or incorrect topic is chosen at random from the ImageNet dataset based on the status of the topic background in the fusion item. In addition, to obtain the cell text, if the status of the text group in the fusion item is correct, a group text is extracted randomly from the text-based CAPTCHA’s text groups. In contrast, three to five characters are chosen at random from MINST for this cell text. The cell text is then distorted and generated into the text image. To obtain the stylized image, the fusion machine merges the text image with the cell topic image to produce the content image, which is then style transferred from the style image. To deceive CNN/DNN networks, the stylized image is transformed into an adversarial example, known as a blended image.
- Finally, the grid-based CAPTCHA is created by combining nine blended image cells.

### 5.4. Cognitive-Based CAPTCHA

**Figure 10:**
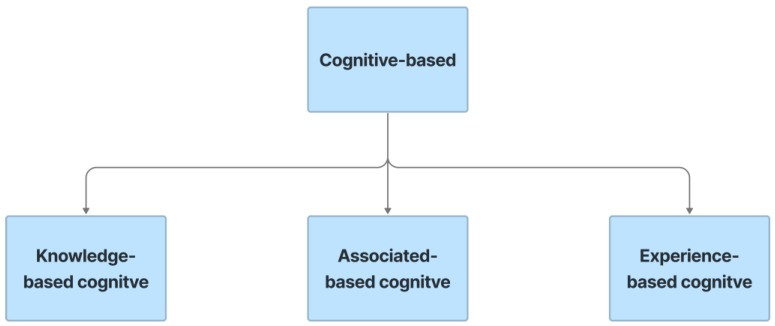
Cognitive-based types.

Cognitive-based CAPTCHA refers to CAPTCHA that is based on cognitive abilities. Cognitive talents are brain-based abilities that result from a unique blend of neurobiological and psychological techniques. Human cognition and behavior include knowledge, concentration, memory, judgment and appraisal, reasoning and calculation, problem solving, and decision making. These CAPTCHA approaches use biometric (something you are), physical (something you have), and knowledge-based (something you know) variables with or without the assistance of sensors such as a gyroscope or accelerometer to discriminate between humans and bots. In this work, the cognitive-based CAPTCHA is built by embedding the cognitive characteristics of knowledge, associated, and experience to the whole CAPTCHA, depicted in Figure 10.

#### 5.4.1. Knowledge-Based

Knowledge-based CAPTCHA requires users to have specific knowledge during verification processes. Typically, in this solution, users need to recognize correct text or synonym text along with correct topic backgrounds and understand the correct order mentioned in the CAPTCHA’s description, such as normal order, inverse order, a special order, etc. This knowledge requires users to be trained or self-taught, which is difficult for automated bots. Furthermore, random combinations of correct text and incorrect topic backgrounds, or incorrect text and correct topic backgrounds, require users to be able to combine many features together, which are difficult for bots to recognize.

#### 5.4.2. Associated-Based

Associated-based CAPTCHA requires users to not only have specific knowledge but also to be able to link their familiarity with visual parts of the CAPTCHA during verification processes. Users are typically required in this solution to link background images to an event (e.g., wedding, Christmas time, etc.), object (e.g., house, car, etc.), or location (e.g., sea, country, etc.) mentioned in the description section.

#### 5.4.3. Experience-Based

Cognitive experience is a psychological foundation for intellectual giftedness and a form of representation (i.e., how an individual sees, understands, and interprets what is occurring in the surrounding reality). It is a proto phenomenon of a person’s intellectual life, the smallest units of conscious experience that are hypothesized and investigated using coordinated phenomenology and neuroscience. In this solution, a user must understand when to select correct or incorrect image cells based on the hint symbol’s signal (e.g., yellow for correct input, red for incorrect input, etc.). Furthermore, after the user selects an image cell, except for the selected cells, other cells are replaced by new images and the locations of all image cells are randomly changed. As a result, users must continuously pay attention to the signal of the hint symbol until the challenge is completed. This feature is also useful in the case of relay attacks.

### 5.5. Security Evaluation

**Figure 11:**
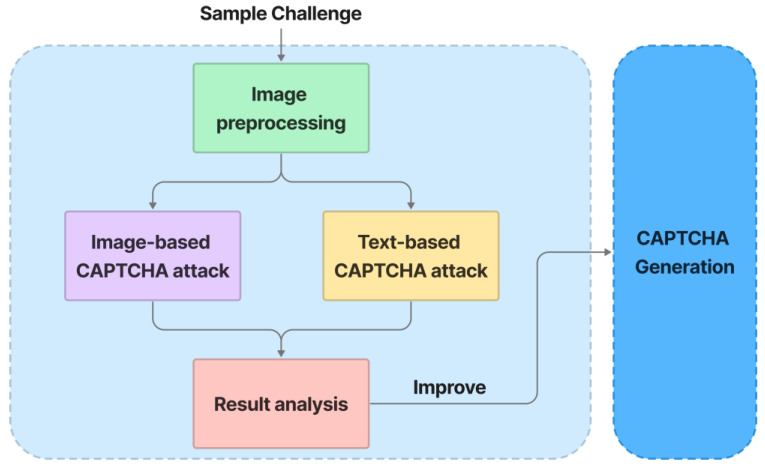
Security evaluation.

Figure 11 depicts the security evaluation process. A sample challenge is preprocessed using Patchwise PCA, JPEG Compression, and Soft-Thresholding methods, which achieve high defense results against adversarial examples in [76]. Following preprocessing, the output is analyzed for text recognition using state-of-the-art models LENET-5 [77] and ResNet-50 [56]. For image classification, the preprocessing output is evaluated using state-of-the-art models VGG-16 [51] and ResNet-101 [56]. Furthermore, the algorithms are designed as modules, making it simple to incorporate new models into the security evaluation process. The analysis results are then used to improve the CAPTCHA-generation process so that it remains strong against automated bots.

### 5.6. Usability Evaluation

**Figure 12:**
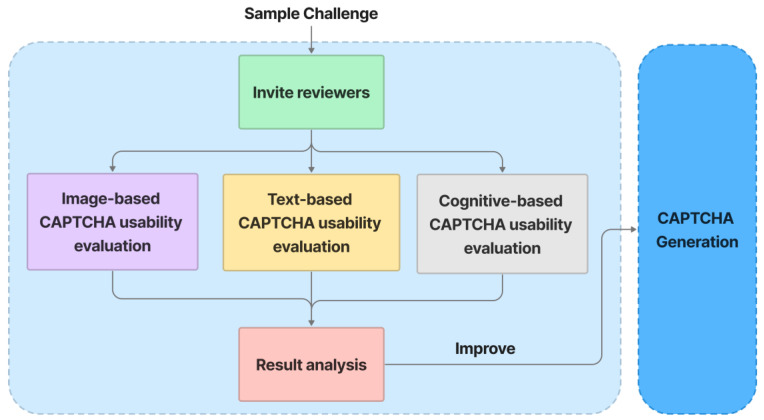
Usability evaluation.

In Figure 12, the usability evaluation process is illustrated as follow:

- Invite involved testers to evaluate a sample challenge.
- Testers evaluate image-based CAPTCHA sections based on how easily the background images can be classified in terms of the overall topic.
- Text-based CAPTCHA sections are evaluated by testers based on how easily they can recognize text.
- The cognitive-based CAPTCHA sections are then evaluated by testers based on how easily a user can interact with CAPTCHA during the resolving time.

The results of the analysis are used to improve the CAPTCHA-generation process, which aims to improve the usability of the CAPTCHA.

## 6. Experiments

### 6.1. Experiment Setup

All experiments were run on a workstation equipped with an Intel(R) Xeon(R) CPU @ 2.30 GHz with 16 GB of RAM, and an NVIDIA TESLA T4 GPU with 16 GB of RAM. The employed deep-learning frameworks are TensorFlow [78] and Torch [79], and the dataset we used is based on Section 5.1.

### 6.2. Usability Analysis

#### 6.2.1. Methodology

**Figure 13:**
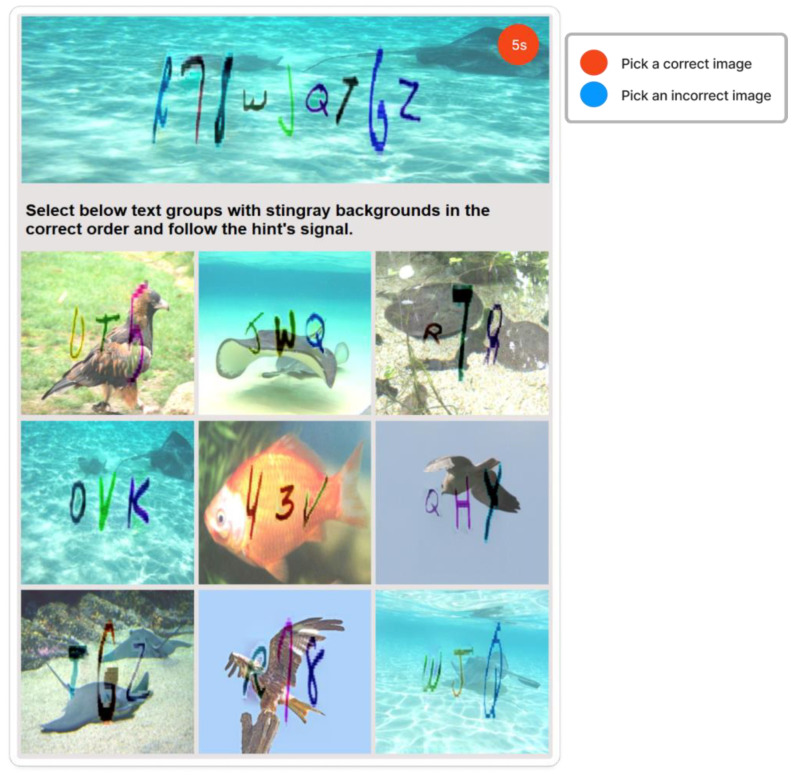
Real example of zxCAPTCHA.

To carry out our evaluation, we built a prototype that testers could use to test the CAPTCHA, shown in Figure 13, and collect evaluation data. Then, from our institution, we recruited volunteer users to conduct the evaluation.

#### 6.2.2. Analysis

**Figure 14:**
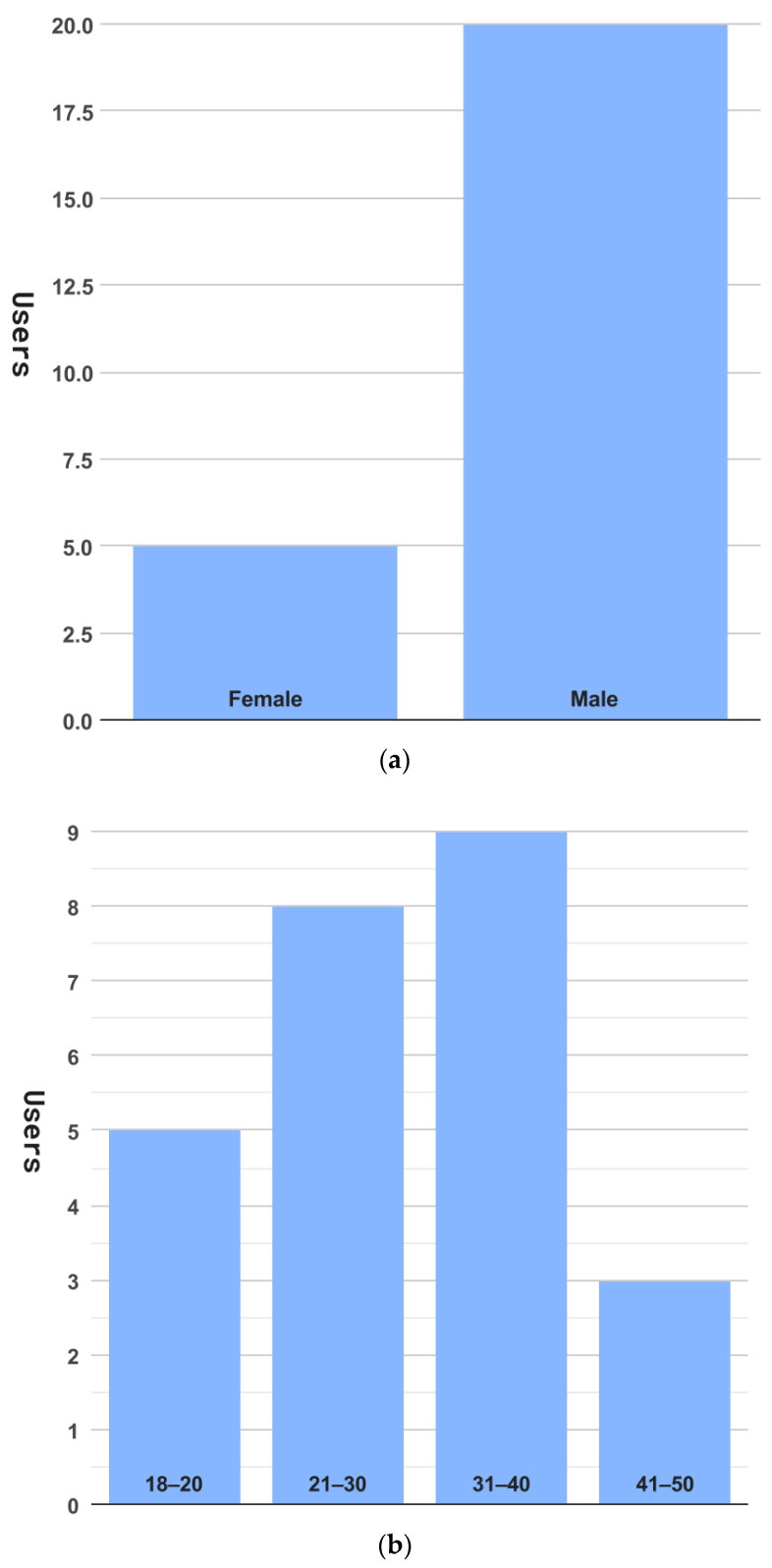

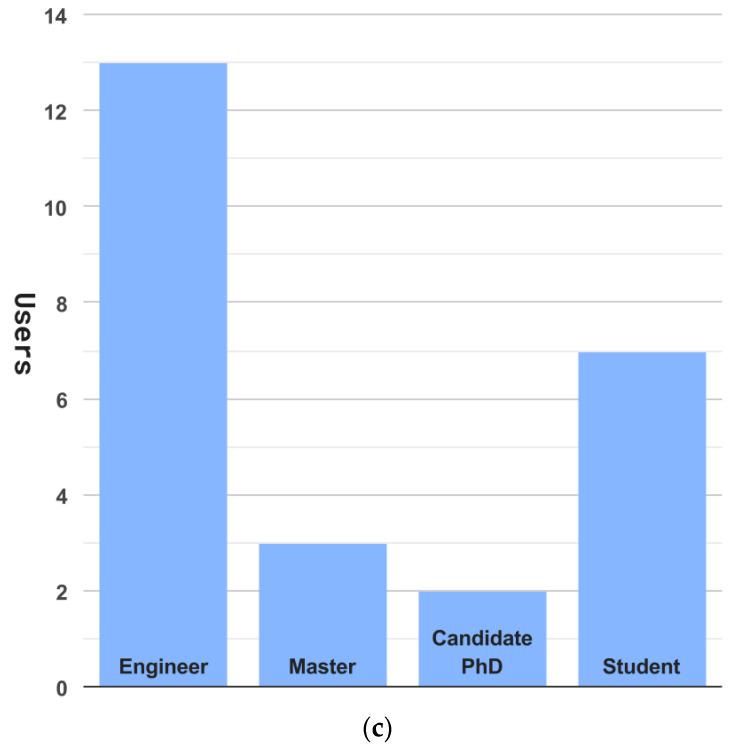
User distribution, (**a**) by genders, (**b**) by ages, and (**c**) by education.

As shown in Figure 14, we recruited 25 volunteer users. There were 5 female users and 20 male users. Most of them were between the ages of 18 and 50 years. Furthermore, almost all the users had a university education or higher. In the evaluation process, all 25 users completed the evaluation. We show the major results based on the collected data in Table 2, where average time, median time, and success rate measure average resolving time, median resolving time, and the average successful probability of all users to complete the relevant activity, respectively.

We tested eight CAPTCHA versions in this study: normal, normal cognitive, stylized, stylized cognitive, adversarial, adversarial cognitive, stylized adversarial, and stylized adversarial cognitive (zxCAPTCHA). Not only do the stylization, adversarial, and cognition versions significantly improve security performance, but also their recognition success rate remains high, although considerably lower than the normal versions. Meanwhile, users take comparable time to resolve stylized adversarial versions and normal versions, but the cognitive versions take longer. This is easy to understand because the cognitive versions require users to pay attention throughout the challenge and users do not get familiar with this CAPTCHA. These findings indicate that zxCAPTCHA and standard CAPTCHAs have comparable usability. Furthermore, we discovered that long text-based CAPTCHAs take longer to recognize and have a lower rate of success than shorter ones. This suggests that there is a balance between security and usability.

### 6.3. Security Analysis

#### 6.3.1. Methodology

In this part, we examined CAPTCHA security in the following ways:

- We assessed the effects of random guess and relay attacks on the CAPTCHA.
- We assessed the ability of some state-of-the-art CNN/DNN networks in Section 5.5 to recognize generated adversarial stylized images and text from the ImageNet and EMNIST datasets.

#### 6.3.2. Analysis

1. Random Guess Attack

This CAPTCHA contains six incorrect image cells (either a wrong background image or incorrect text) and three correct image cells. Users must choose both correct and incorrect image cells based on a signal from the hint. In a challenge, the selection times range from five to nine, implying that a user must select at least five random image cells to complete the challenge. Hence, the probability ***P*** in random guessing is extremely small:

$$P_{2}\leq P\leq P_{1}$$

with:

$$\begin{array}{c} P_{1}=\frac{1}{9}\times \frac{1}{8}\times \frac{1}{7}\times \frac{1}{6}\times \frac{1}{5}\approx 0.000066\\ P_{2}=\frac{1}{9}\times \frac{1}{8}\times \frac{1}{7}\times \frac{1}{6}\times \frac{1}{5}\times \frac{1}{4}\times \frac{1}{3}\times \frac{1}{2}\approx 0.00000275 \end{array}$$

2. Relay Attack

**Figure 15:**
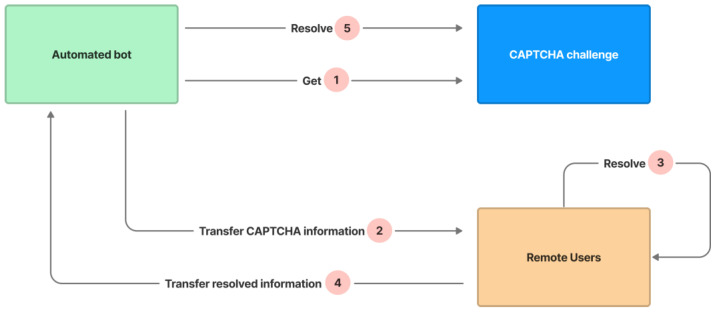
Relay attack steps.

CAPTCHAs are designed to be completed by humans, but there are markets for labor services solving CAPTCHAs (usually in low-wage areas) and relay attacks that send CAPTCHA challenges to humans who benefit from solving them. The attack is depicted in Figure 15:

- An automated bot gets a challenge from the CAPTCHA in step 1.
- The bot transfers the CAPTCHA’s information to a remote user in step 2.
- The remote user resolves the CAPTCHA by recognizing the text and indicating correct or incorrect image cells in step 3.
- The remote user transfers the resolved information to the bot through API in step 4.
- The bot resolves the CAPTCHA’s challenge based on the remote user’s resolved information in step 5.

The CAPTCHA avoids the attack by changing the signal attributes (such as the signal colors for picking correct or incorrect image cells) of the hint five seconds per time and changing the positions and contents of unpicked image cells after any image cell is picked. The automated bot must regularly imitate the complicated function of clicking on the hint symbol to obtain the hint information and send the CAPTCHA challenge information to the assisting remote user, and the remote user must pay attention and resolve the challenge many times. As a result, this necessitates a complicated mechanism for communication between bots and remote users, which causes the bot’s resolving time to exceed the CAPTCHA’s allowed resolving time.

3. Adversarial and Style Transfer

a. Text-based Evaluation

This evaluation process employs different state-of-the-art networks (Section 5.5), LeNet-5 and ResNet-50, to recognize four test datasets from the EMNIST Digits dataset (normal, stylized, adversarial, and stylized adversarial), each containing the same 1000 blended images from 10 categories of characters, each with 100 testing images. The selection networks are trained using the selection character image set (Section 5.1), which contains 10,000 hand-labeled images from 10 different categories, each with 1000 images.

In practice, automated bots need to address the positions of each character in images before recognizing them. They also face many difficulties in correctly addressing each character’s position in cluttered image backgrounds; however, we do not include this in this section. In this case, the evaluation analyzes CNN/DNN networks’ ability to recognize each character with different versions, such as normal, stylized, adversarial, and stylized adversarial versions. Table 3 depicts a real sample in the evaluation process.

Table 4 shows the FGSM algorithm’s security performance (Section 4.2.1), while Table 5 shows the BPDA algorithm’s security performance (Section 4.2.2). The results show that stylized adversarial versions are resistant to attacks on state-of-the-art networks. In normal versions, the networks LeNet-5 and ResNet-50 can attain a very high success rate, greater than 95%, but in stylized adversarial versions, their success rates are very low, less than 25%.

b. Image-based Evaluation

This evaluation process employs different state-of-the-art networks (Section 5.5), VGG-16 and ResNet-101, to recognize four test datasets (normal, stylized, adversarial, and stylized adversarial), each containing the same 1000 blended images from 100 categories, each with 10 testing images, from the ImageNet dataset (Section 5.1). The selection networks are trained on the selection image set (Section 5.1), containing 10,000 user-friendly hand-labeled images from 100 classes, with 100 images in each. Table 6 depicts a real sample in the evaluation process.

We performed evaluations on normal versions, using original images from the ImageNet dataset and random characters from EMNIST (Section 5.1) with distortion optimization [4.3]. The same evaluations were performed on stylized versions implemented by the neural-style-transfer technique (Section 4.1), adversarial example versions implemented by the adversarial example technique (Section 4.2), and blended versions of the stylization and adversarial example techniques. Character lengths *l*, ranging from three to five, were also studied for security performance. Table 7 depicts the level of security of the BPDA algorithm (Section 4.2.2), whereas Table 8 depicts the security performance of the FGSM algorithm (Section 4.2.1). These result tables show that image stylization still influences the recognition of neural networks more than normal versions, whereas adversarial examples clearly have a considerably great effect on fooling the neural networks. As a result, combining stylization and adversarial examples yields the strongest version with the highest fooling rate on neural networks (success recognition rates lower than 45%). Furthermore, the result proved that the longer the text, the higher the rate of deception on neural networks.

## 7. Conclusions

In our study, we introduced zxCAPTCHA, a CAPTCHA that combines the characteristics of text-based, image-based, and cognitive-based CAPTCHA schemes, as well as adversarial examples, neural style transfer, and defense deep-learning methods, to improve the security of the CATCHA. Unlike previous research of CAPTCHA in which designers only presented changes by attaching artificial noise, distortion, or a complex background, this proposed CAPTCHA is based on deep-learning and cognitive techniques. Extensive security and usability evaluations are performed to determine the effectiveness of these techniques in improving CAPTCHA security. The results demonstrate that the developed CAPTCHA enhances the security of standard CAPTCHAs while maintaining comparable accessibility. The proposed CAPTCHA can be developed for different cognitive CAPTCHA variations by changing their attributes. Specifically, the attribute of text group order could be natural order, inverse order, or a special order, such as any text group with special characteristics requested to be picked up with higher priority. Furthermore, the background images and text can be localized to make them more familiar to users in their local surroundings. The hint signals will be an interesting part, challenging users’ cognition. Currently, the study is focusing on picking correct or incorrect images. However, hint signals can focus on other cognition aspects, such as picking images with specific background objects. As a result, we can see that the proposed CAPTCHA is simple to adapt to any system that requires CAPTCHA protection against automated bots.

To summarize, we expect that this work will serve as a good starting point for new CAPTCHA design for the development of new secure CAPTCHA schemes. Second, because the results of this study show that deep learning combined with cognitive techniques can strongly improve CAPTCHA security, it would be advantageous to implement the proposed CAPTCHA for security enhancement for systems in practice, and this will point the way forward for future CAPTCHA research.

## Figures and Tables

**Table 1:** The list of distortions.

Distortion Type	Category	Level	Intensity Range
Rotation	Geometric	1	45–135
Width scaling		1	1.5–3
Height scaling		1	1.5–3
Piecewise scaling		2	1.5–3
Shadow		2	2–5
Outline		1	1–10
Striped		1	45–135
Tilting		1	1–10
Erosion	Degradation	2	3–3.5
Grains		2	1–10
Random outline degradation		2	1–10
Periodic noise	Noise	1	5–7
Salt and pepper noise		1	10–20
Speckle noise		1	2–5

**Table 2:** Usability result.

Factor	Normal	Normal Cognitive	Stylized	Stylized Cognitive	Adversarial	Adversarial Cognitive	Stylized Adversarial	Stylized Adversarial Cognitive
Success rate	88%	80%	84%	72%	88%	76%	84%	76%
Average time	12.7 s	15.2 s	12.3 s	14.9 s	11.8 s	15.7 s	12.5 s	15.6 s
Median time	9.5 s	13.1 s	10.5 s	12.5 s	9.3 s	13.5 s	10.7 s	12.3 s

**Table 3:** Real sample for text evaluation.

Style	Normal	Stylized	Adversarial	Stylized Adversarial
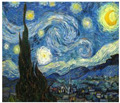	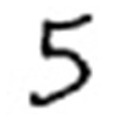	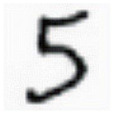	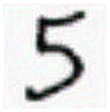	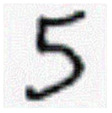

**Table 4:** Character-based FGSM security performance.

Recognition Network	Normal	Stylized	Adversarial		Stylized Adversarial	
			Generated by VGG-16	Generated by ResNet-101	Generated by VGG-16	Generated by ResNet-101
LeNet-5	95.7	37.6	17.3	19.7	7.8	9.3
ResNet-50	97.5	43.6	25.5	28.1	15.8	17.3

**Table 5:** Character-based BPDA security performance.

Recognition Network	Normal	Stylized	Adversarial		Stylized Adversarial	
			Generated by VGG-16	Generated by ResNet-101	Generated by VGG-16	Generated by ResNet-101
LeNet-5	95.7	37.6	23.3	25.6	11.3	13.6
ResNet-50	97.5	43.6	31.1	32.5	20.3	23.5

**Table 6:** Real sample for image evaluation.

Style	Normal	Stylized	Adversarial	Stylized Adversarial
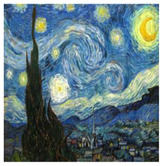	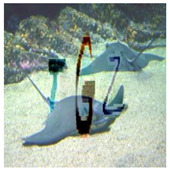	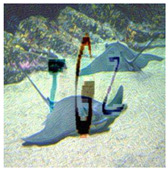	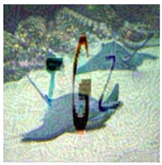	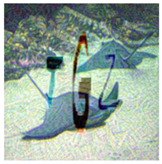
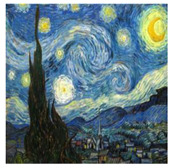	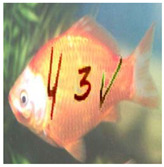	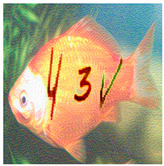	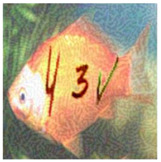	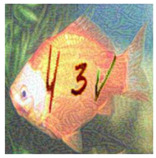
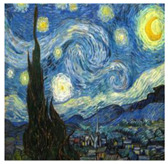	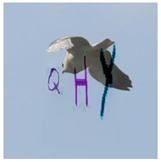	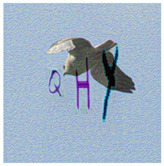	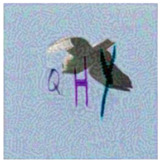	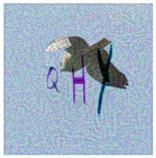
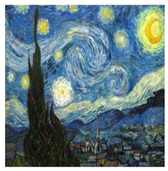	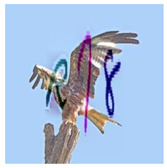	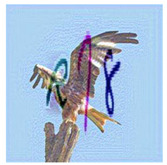	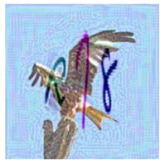	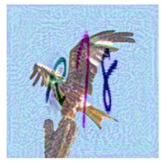
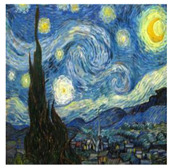	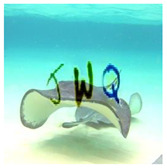	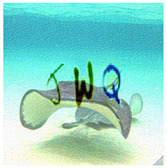	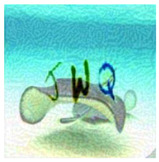	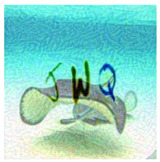

**Table 7:** Image-based BPDA security performance.

Recognition Network	Normal			Stylized			Adversarial						Stylized Adversarial					
							Generated by VGG-16			Generated by ResNet-101			Generated by VGG-16			Generated by ResNet-101		
	l = 3	l = 4	l = 5	l = 3	l = 4	l = 5	l = 3	l = 4	l = 5	l = 3	l = 4	l = 5	l = 3	l = 4	l = 5	l = 3	l = 4	l = 5
VGG-16	78.5	77.3	76.7	61.6	61.3	60.1	48.7	46.3	45.7	54.2	53.1	52.3	35.1	34.7	34.5	40.3	39.2	37.4
ResNet-101	87.1	85.3	85.1	70.5	68.3	67.7	58.3	56.1	54.6	60.4	58.7	56.2	43.2	41.5	42.1	44.7	43.5	42.3

**Table 8:** Image-based FGSM security performance.

Recognition Network	Normal			Stylized			Adversarial						Stylized Adversarial					
							Generated by VGG-16			Generated by ResNet-101			Generated by VGG-16			Generated by ResNet-101		
	l = 3	l = 4	l = 5	l = 3	l = 4	l = 5	l = 3	l = 4	l = 5	l = 3	l = 4	l = 5	l = 3	l = 4	l = 5	l = 3	l = 4	l = 5
VGG-16	78.5	77.3	76.7	61.6	61.3	60.1	35.6	33.3	31.5	41.3	40.3	39.7	27.3	25.6	23.7	33.4	31.8	31.5
ResNet-101	87.1	85.3	85.1	70.5	68.3	67.7	45.3	43.6	42.7	47.1	46.3	45.7	35.3	33.5	31.8	37.1	36.5	35.7

## Data Availability

The datasets generated during and/or analyzed during the current study are available from the corresponding author upon reasonable request.

## Abbreviations

CAPTCHA	Completely Automated Public Turing test to tell Computers and Humans Apart
HIP	Human Interactive Proof
EMNIST	Extended Modified National Institute of Standard and Technology
CNN	Convolutional Neural Network
DNN	Deep Neural Network
ML	Machine Learning
CI/CD	Continuous Integration and Continuous Delivery
FGSM	Fast Gradient Sign Method
BPDA	Backward Pass Differentiable Approximation
VGG	Visual Geometry Group
ResNet	Residual Neural Network
CV	Computer Vision
OCR	Optical Character Recognition
IAN	Immutable Adversarial Noise
GAN	Generative Adversarial Network
SVM	Support Vector Machine
